# Round about the Asylums.—XIII

**Published:** 1888-08-25

**Authors:** 


### ROUND ABOUT THE ASYLUMS.—XIII.

BY A MEDICAL SUPERINTENDENT.

### LANCASHIRE ASYLUM AT PRESTWICH.

HERE we come to the largest asylum in England. On the last day of the year there were 2,307 patients resident, and nearly one thousand were admitted during the preceding twelvemonths; 619 were discharged, either recovered or relieved, and 210 died. The recoveries stand at 34 per cent. on the admissions, and the deaths at 9 per cent. on the average number resident. The weekly cost of maintenance was 7*s.* 9½*d.* per head. Private patients are charged from 15*s.* to 21*s.* a week, but there are only 35 of this class in the asylum. When commenting on the admissions, Mr. Ley remarks that there is "a growing tendency on the part of workhouse officials to rid themselves of the trouble of managing imbecile children, and looking after old people in their second childhood. These latter cases are certainly troublesome to manage, but unfortunately the asylum can do them little good." We are not so sure of this, and we refer Mr. Ley to the Lichfield Asylum report for Dr. Spence's view of the same question. Mr. Ley continues, "it has often been my experience to observe unpleasant results from the presence of imbecile children among the adult insane." This bad influence is an undoubted fact, and yet very few counties have built accommodation specially for idiot children; indeed, we believe no separate wards are anywhere provided, except at the Northampton County Asylum. We noticed in the Rainhill report that the large annexe was almost empty. Why not fit it, or part of it, as an idiot asylum, and so meet the want which Mr. Ley so much deplores? The Commissioners say that the percentage of patients employed is high, being 73 for the men, and 81 for the women. Referring to the causes of insanity in those admitted during the year, we find that hereditary influence accounted for 256, and drink for 226. Fifty-six of the deaths were owing to some form of lung disease.

### LANCASHIRE ASYLUM AT LANCASTER MOOR.

This is another Leviathan, but in comparison with Prestwich it is almost small, for it contained at the end of the year 1,597 patients. The recoveries were 35·8 per cent. on the admissions, and the deaths were 9·75 on the average number resident. Dr. Cassidy notes an outbreak of dysentery, from which twelve patients died. The cause of the outbreak could not be found, and it is a little curious that most of the cases occurred in the annexe, which is almost new, and in which the sanitary arrangements are presumably as nearly perfect as possible. We are surprised to learn that this large asylum does not possess a detached hospital for the treatment of infectious diseases. The Commissioners say that the wards generally were in very good order. The weekly cost of maintenance is 7*s.* 0⅝*d.* per head.

### MIDDLESEX COUNTY ASYLUM AT BANSTEAD.

Seven hundred and thirteen men and 1,285 women were resident at the end of the year, making a total of just 2 under 2,000 patients. Four hundred and twenty-seven patients were admitted, and the recoveries calculated on the admissions were 34·66 per cent. Two hundred and forty patients died, being at the rate of 11·90 per cent. on the average number resident. Sixty-one of the deaths were due to consumption, 51 to general paralysis, and upwards of 70 to other brain diseases. The weekly rate of maintenance stands at the same as Hanwell—9*s.* 7½*d.* The Commissioners say that 69 per cent. of the males and only 41 per cent. of the females are usefully employed; and they add that they "thought the provision of light literature, games, etc., in the wards somewhat scanty."

